# P-1499. Evaluating the Long-Term Antibody Response of MVA-BN in PLWH: A Two-Year Follow-Up Study

**DOI:** 10.1093/ofid/ofaf695.1683

**Published:** 2026-01-11

**Authors:** Pierluigi Francesco Salvo, Francesca Lombardi, Valentina Iannone, giulia lenzi, Rebecca Jo Steiner, Andrea Carbone, Carlo Torti, Simona Di Giambenedetto, Gianmaria Baldin

**Affiliations:** Università Cattolica del Sacro Cuore, Roma, Lazio, Italy; Fondazione Policlinico A. Gemelli IRCCS, roma, Lazio, Italy; Università Cattolica del Sacro Cuore, Roma, Lazio, Italy; Università Cattolica del Sacro Cuore, Roma, Lazio, Italy; Università Cattolica del Sacro Cuore, Roma, Lazio, Italy; Università Cattolica del Sacro Cuore, Roma, Lazio, Italy; Università Cattolica del Sacro Cuore, Roma, Lazio, Italy; Università Cattolica del Sacro Cuore, Roma, Lazio, Italy; Fondazione Policlinico A. Gemelli IRCCS, roma, Lazio, Italy

## Abstract

**Background:**

Mpox has been reclassified as a Public Health Emergency of International Concern, highlighting the urgent need for effective vaccination strategies. PLWH may have an altered immune response, raising concerns about the efficacy of vaccines such as MVA-BN, which has shown promise in the general population. We conducted this study to evaluate the antibody response to the MVA-BN vaccine in PLWH

**Table 1:**
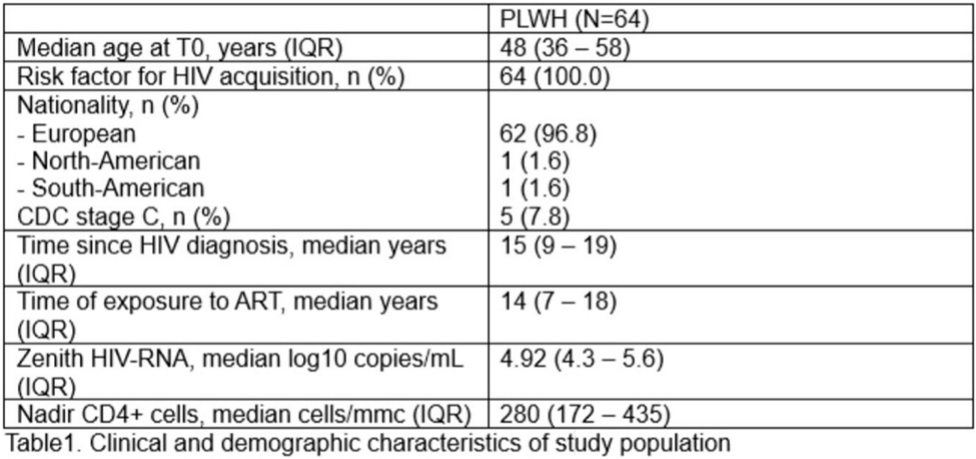
Clinical and demographic characteristics of study population

**Methods:**

From August 2022 to November 2024, serum samples were collected from PLWH attending our outpatient clinic and who were vaccinated with the MVA-BN. IgG against mpox were measured on cryopreserved serum samples by ELISA. No cross-reactivity or interference between anti-mpox IgG and analogues was reported. The MPXV-specific IgG levels were determined at several time points: immediately prior to vaccination (T0), immediately following the vaccine completion (within 6 months, T1), at 12 months (T2), and 24 months after immunization (T3). We used an adjusted linear mixed model for repeated measures to evaluate the effects of vaccination on the IgG levels over time

**Results:**

A total of 64 PLWH were enrolled. Characteristics of the participants are summarized in Tabl1. In the unadjusted analysis, no statistically significant differences in IgG levels were observed between time points. Following adjustment for potential confounders – namely, age, zenith of HIV-RNA, nadir of CD4 cells – the mixed effects model revealed an upward trend in antibody levels only at T1 compared to T0 (p=0.004). This suggests a slight increase in the immune response immediately after vaccination, which both at T2 and T3 reverted to levels comparable to T0. Notably, one participant with a constant trend over time, exhibited an exponential increase in IgG levels at T3. The participant reported a single vesicular lesion in the oral cavity, several days prior to the T3 blood sample collection.

**Conclusion:**

The findings suggest that MVA-BN can elicit only a moderate and not prolonged immune response in PLWH. However, the significant antibody peak observed in one participant, despite the presence of very mild symptoms, highlights the potential for MVA-BN, in the case of overlapping natural infection, to provide robust hybrid protection even in this vulnerable population. Further research is needed to assess the role of cellular immunity.

**Disclosures:**

All Authors: No reported disclosures

